# Exercise mediates myocardial infarction via non-coding RNAs

**DOI:** 10.3389/fcvm.2024.1432468

**Published:** 2024-11-01

**Authors:** Changliang Han, Cuili Zhai, Ailing Li, Yongzhi Ma, Jamal Hallajzadeh

**Affiliations:** ^1^ Lishui University, Lishui, Zhejiang, China; ^2^ College of Chinese Martial Arts, Beijing Sport University, Beijing, China; ^3^ City University of Malyasia, Kuala Lumpur, Malaysia; ^4^ Division of Sports Science and Physical Education, Tsinghua University, Beijing, China; ^5^ Department of Biochemistry and Nutrition, Research Center for Evidence-Based Health Management, Maragheh University of Medical Sciences, Maragheh, Iran

**Keywords:** exercise, myocardial infarction, non-coding RNAs, cardioprotection, apoptosis

## Abstract

Myocardial infarction (MI), a widespread cardiovascular issue, mainly occurs due to blood clot formation in the coronary arteries, which reduces blood flow to the heart muscle and leads to cell death. Incorporating exercise into a lifestyle can significantly benefit recovery and reduce the risk of future cardiac events for MI patients. Non-coding RNAs (ncRNAs) play various roles in the effects of exercise on myocardial infarction (MI). ncRNAs regulate gene expression, influence cardiac remodeling, angiogenesis, inflammation, oxidative stress, apoptosis, cardioprotection, and cardiac electrophysiology. The expression of specific ncRNAs is altered by exercise, leading to beneficial changes in heart structure, function, and recovery after MI. These ncRNAs modulate molecular pathways that contribute to improved cardiac health, including reducing inflammation, enhancing angiogenesis, promoting cell survival, and mitigating oxidative stress. Furthermore, they are involved in regulating changes in cardiac remodeling, such as hypertrophy and fibrosis, and can influence the electrical properties of the heart, thereby decreasing the risk of arrhythmias. Knowledge on MI has entered a new phase, with investigations of ncRNAs in physical exercise yielding invaluable insights into the impact of this therapeutic modality. This review compiled research on ncRNAs in MI, with an emphasis on their applicability to physical activity.

## 1 Introduction

MI, also known as heart attack, is defined as the death of heart muscle cells due to a prolonged lack of oxygen supply. MI refers to the irreversible damage and tissue death caused by a critical reduction in blood flow and oxygen delivery to a region of the heart (1). MI is a substantial global concern that is linked to a high death rate and substantial heart injury (2). Currently, standard treatments, such as medication, surgery, and interventional procedures, are essential for the management of MI. The most effective treatment for MI involves the restoration of blood flow to the affected area of the heart as quickly as possible. While reperfusion therapies like percutaneous coronary intervention (PCI) and thrombolytic drugs have greatly improved outcomes for patients with acute MI by re-establishing blood flow, the incidence of heart failure following MI remains a major clinical issue (3, 4). Cardiac rehabilitation, however, is the final component of post-MI treatment and is crucial for increasing patient satisfaction and lowering death rates (5).

**Figure 1:**
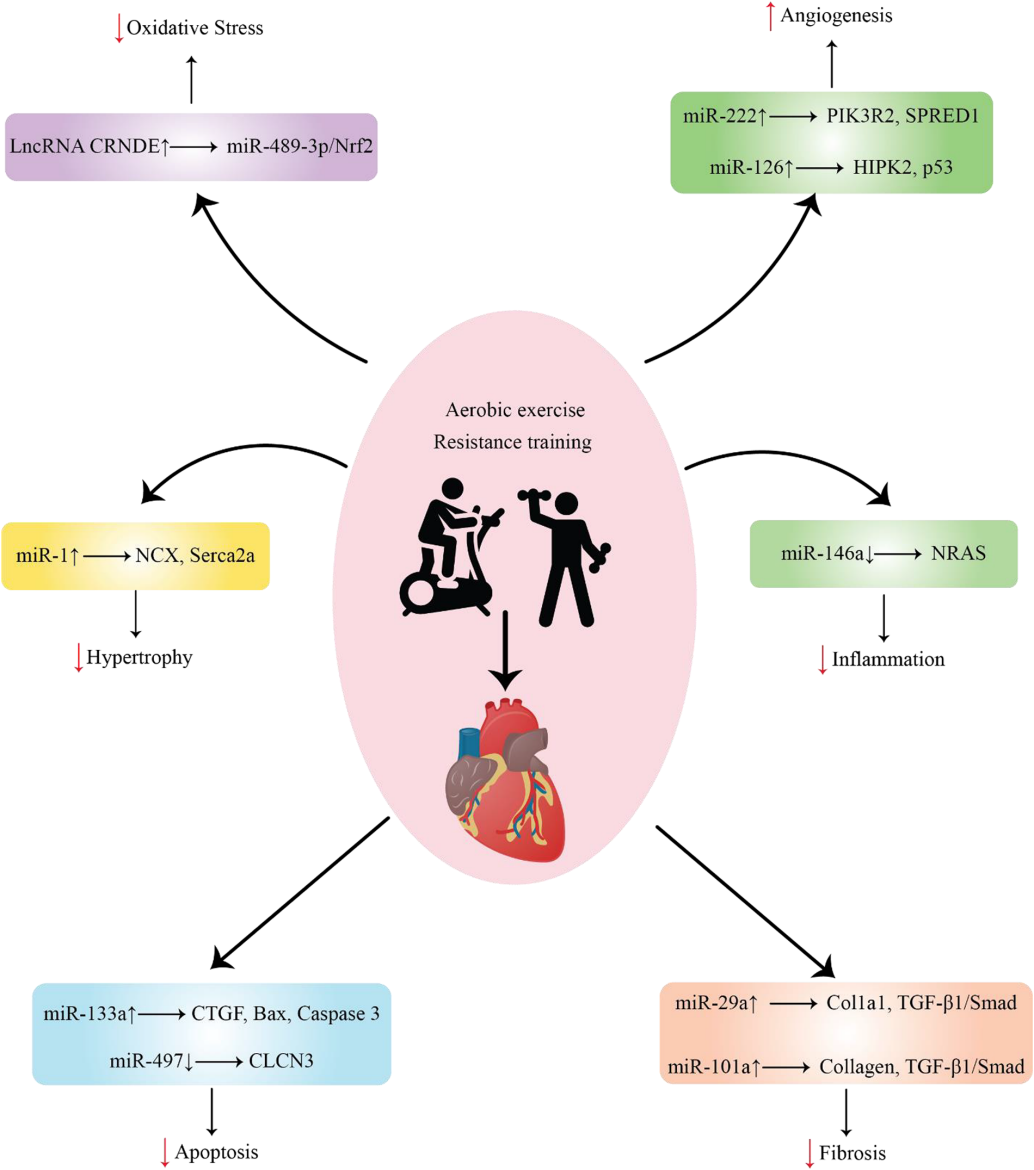
Representation of various ncRNAs released by cells in response to exercise after MI and their function.

The abundance of advantages of exercise regimens over sedentary behavior has progressively been validated, leading to their emergence as a treatment option for individuals afflicted with several chronic illnesses, including tumor malignancies, fatty liver, stroke, type II diabetes, multiple sclerosis, and cardiovascular disease (CVDs) (6–10). Exercise training is an essential component of cardiac rehabilitation programs for patients who have experienced a MI or other types of heart disease (5, 11, 12). With a growing body of evidence supporting exercise training's preventive effects against many cardiovascular illnesses, exercise training has been accepted as a viable strategy for actively assisting in rehabilitating hearts after MI (Figure 1) (13). A natural approach to heart rehabilitation is an exercise that does not require drugs. Scholars and medical professionals have indeed placed great emphasis on the preventive and cost-effective benefits of exercise as a rehabilitation strategy for patients with heart disease, particularly after MI or other cardiac events. However, the exact mechanism underlying myocardial protection against ET-induced MI remains unclear.

A broad class of functional RNA molecules that cannot be coded as proteins are referred to as ncRNAs. They can be long non-coding RNAs (lncRNAs) with more than 200 nucleotides or small microRNAs (miRNAs) with less than 22 nucleotides (14, 15). miRNAs are a subclass of short nRNAs that mainly target the 3′-untranslated regions (3′-UTR) of target mRNAs. They have a negative effect on post-transcriptional gene regulation. Numerous biological processes, including angiogenesis, differentiation, migration, apoptosis, and proliferation, are influenced by microRNAs (16–18). LncRNAs act as molecular sponges for other RNA molecules, such as miRNAs. By acting as molecular sponges, lncRNAs can indirectly regulate gene expression by modulating miRNA availability and activity. Dysregulation of lncRNA-mediated competitive endogenous RNA (ceRNA) interactions is associated with various diseases, including cardiovascular disorders. Circular RNAs (circRNAs) are a type of ncRNA that form a closed-loop structure. Thousands of circRNAs have been discovered in numerous species, exhibiting varying levels of abundance across different cell types and in biological fluids. CircRNAs exert different biological functions in the cells by acting as transcriptional regulators and miRNA sponges, as well as serving as protein decoys, scaffolds and recruiters (19).

The presence of ncRNAs in extracellular vesicles (EVs) has become a significant area of research, highlighting the complex roles these molecules play in intercellular communication and various biological processes. EVs, such as exosomes, microvesicles (MVs), and apoptotic bodies, are gaining attention for their significant role in facilitating communication between cells in cardiovascular diseases (20, 21). EVs are released by various cell types, including cardiomyocytes (CMs), endothelial cells, and cardiac fibroblasts (CFs), and they help safeguard the ncRNA cargo from degradation. Notably, mounting data suggests that target genes and related signaling pathways are extensively regulated by ncRNAs and exosomal ncRNAs, especially miRNAs, which may contribute to various beneficial effects of exercise on the heart (22–25). This suggests that ncRNAs could serve as helpful therapeutic targets, reducing the beneficial effects of exercise on CVD prevention. This article provides an overview of the protective effects of physical exercise against MI and the roles of ncRNAs in this process.

## 2 ncRNAs

Non-coding RNAs (ncRNAs), which are small RNA molecules that do not code for proteins, make up the majority of RNA transcripts found in cells. This diverse category of endogenous RNA includes short microRNAs (miRNAs), approximately 22 nucleotides in length, as well as long non-coding RNAs (lncRNAs), which are over 200 nucleotides long (14, 15). miRNAs are essential for the post-transcriptional regulation of gene expression. They primarily operate by binding to complementary regions of target mRNAs, which can either inhibit translation directly or facilitate mRNA degradation. This interaction, which depends on specific sequences, enables miRNAs to precisely adjust a variety of biological processes. The maturation of miRNAs requires a complex and extensive network that coordinates the transcription of pri-miRNAs. This process includes cleavage by endonucleases and the transport of miRNAs from the nucleus to the cytoplasm, followed by additional cleavage and integration into the RNA-induced silencing complex (RISC). Within this complex, miRNAs can either inhibit the translation of target gene mRNAs or promote their degradation by base-pairing with the 3′ untranslated region (3′ UTR) (16–18). Increasing evidence indicates that miRNAs are crucial in adapting to exercise. These molecules affect muscle responses to physical activity by regulating gene expression, supporting muscle growth, and aiding recovery. For example, miR-1 and miR-133 are associated with muscle hypertrophy and endurance adaptations, underscoring their significance in physiological changes. Gaining insight into these mechanisms may lead to better strategies for enhancing athletic performance and rehabilitation. Alongside miRNAs, lncRNAs are also vital in regulating cellular processes. Most lncRNAs are transcribed by RNA polymerase II and act as further regulators of the genome through various mechanisms. As transcriptional regulators, lncRNAs can influence multiple stages of transcription, including initiation, elongation, and termination. They also play a role in post-transcriptional processes, such as splicing, transport, translation, mRNA stability, and subcellular localization. Recent studies have shown that lncRNAs can function as cofactors, competitors, and decoys for RNA-binding proteins and miRNAs. By interacting with these proteins, lncRNAs can either enhance or inhibit their activities, thereby influencing various cellular processes. As competitors, lncRNAs can trap RNA-binding proteins, hindering their ability to engage with target mRNAs. Furthermore, as decoys, lncRNAs can bind to miRNAs, blocking them from silencing their target genes. These diverse functions emphasize the flexible regulatory roles of lncRNAs in gene expression and cellular signaling. CircRNAs are intriguing biomolecules produced from pre-mRNA transcripts through a unique process known as noncanonical back-splicing (26). In this mechanism, a downstream 3′ splice site is linked to an upstream 5′ splice site, resulting in the formation of a closed-loop structure via a 3′–5′ phosphodiester bond (19, 26). Unlike linear mRNAs, circRNAs do not have the typical 5′ cap or 3′ poly(A) tail, contributing to their distinct stability and functionality. Their covalently closed-loop structure makes circRNAs resistant to degradation by common ribonucleases, such as RNase R, which typically target linear RNA molecules (19, 26). Interestingly, circRNAs can be produced not only from mRNA precursors but also from lncRNAs through comparable back-splicing mechanisms. This flexibility in their origins enables a wide variety of circRNA structures and functions (26). These properties enable circRNAs to play various roles in gene regulation, including acting as microRNA sponges (19), interacting with RNA-binding proteins (26), and influencing transcriptional and translational processes (19). Their stability and unique structure make them potential targets for therapeutic applications and biomarkers in various diseases.

## 3 Cardiac hypertrophy in post-MI

Preclinical studies have shown that miRNAs, including miR-132, can affect key signaling pathways involved in various aspects of cardiac function and remodeling during the development of cardiac hypertrophy (27). These miRNAs have been found to regulate signaling pathways that control cardiomyocyte hypertrophy and growth, such as the PGC-1a/NRF2 signaling pathway (28), and dysregulation of miRNA expression can lead to pathological cardiomyocyte enlargement and tissue-level cardiac hypertrophy. In addition, miRNAs can modulate the signaling cascades governing autophagy, which is crucial for maintaining homeostasis and responding to stressors. The disruption of miRNA-mediated autophagy pathway regulation contributes to maladaptive cardiac hypertrophy (29). miRNAs can also target genes involved in intracellular calcium handling, such as SERCA2A, and alterations in calcium homeostasis and signaling due to miRNA dysregulation can impair cardiomyocyte contractility and cardiac function (30). Furthermore, miRNAs have been shown to regulate the expression of genes essential for cardiomyocyte contractility and excitation-contraction coupling, and disruption of these miRNA-mediated mechanisms can lead to changes in cardiac contractile performance during the progression of hypertrophy (30). These preclinical findings highlight the important role of miRNAs, including miR-132, as critical regulators of signaling pathways involved in various aspects of cardiac remodeling and dysfunction associated with pathological cardiac hypertrophy. Antisense therapy is an active research area, and ongoing clinical trials are exploring its use for treating pathological cardiac hypertrophy. Extensive preclinical studies, including large animal models of heart failure, have demonstrated the potential of CDR132l as a first-in-class miR-132 inhibitor (27). These studies showed that CDR132l had a favorable safety profile, was efficiently delivered to cardiac tissue, and showed potent, dose-dependent reduction of miR-132 levels. Preclinical data also indicated that CDR132l treatment promoted improvements in cardiac remodeling and left ventricular function, which are relevant biomarkers of heart failure severity.

## 4 Exercise-induced miRNAs as regulators of hypertrophy in hearts post-MI

A single miRNA can target several genes, whereas many miRNAs can target different genes. Under normal circumstances, miRNAs are believed to primarily regulate gene expression; however, in diseased conditions, their silencing impact becomes more pronounced (31). Most pathogenic events, including RAS-induced cardiac remodeling, are linked to miRNA involvement (32). The regulation of certain miRNAs by exercise can protect against pathological cardiac hypertrophy (Figure 1), which increases the risk of myocardial infarction. It may also promote beneficial remodeling processes in the heart (Table 1).

**Table 1:** Role of exercise via non-coding RNAs in hearts post-MI.

Ref	Type of models	Methodology of exercise	Sample	NcRNAs in heart post-MI	Expression after exercise	Target genes	Modulation
Melo et al. (67)	*In vivo*	5 swimming sessions/week for 10 weeks	Tissue	miR-29	miR-29↑	COL IAI, COL IIIAI	Fibrosis
Melo et al, (35)	*In vivo*	5 ET sessions days/week for 10 weeks	Tissue	miR-1, miR-214	miR-1↑, miR-214↓	NCX, Serca2a, ANF, α-actin	Ventricular hypertrophy
Guo et al. (46)	*In vivo*	5 exercise session/weeks for 4 weeks	Tissue	miR-126↓	miR-126↑	Spred1	Angiogenesis
Wang et al. (92)	In vivo	5 swimming sessions/weeks for 4 weeks	serum	miR-1192	miR-1192↑	Caspase 3, Bax, TGF-β1	Apoptosis
Song et al. (50)	*In vivo*, *in vitro*	5 treadmill sessions/week for 4 weeks	Tissue	miR-126	miR-126↑	PIK3R2, SPRED1	Angiogenesis
Li et al. (91)	*In vivo*	5 treadmill sessions/week for 8weeks	Blood and tissue	miR-497	miR-497↓	CLCN3	Apoptosis, inflammation
Liao et al. (69)	*In vivo*	5 treadmill sessions/week for 2 weeks	Tissue	miR-223-3p, miR-150-5p miR-125b-5p, miR-125b-5p, miR-17-5p	miR-125b-5p↑, miR-17-5p↑, miR-150-5p↑, miR-223-3p↑, miR-125b-5p↑	ITGA1, VTCN1, VDR, TGF-β1, SMAD3, MAPK14, FN1	Angiogenesis, fibrosis, ventricular remodeling, inflammation
Gui et al. (51)	*In vivo*, *in vitro*	5 treadmill sessions/week for 4 weeks	Tissue	miR-126	miR-126↑	Spred1	angiogenesis
Stølena et al. (66)	*In vivo*	5 treadmill sessions/week for 6–8 weeks	Tissue	21 miRNAs	miRNAs ↑ or miRNAs ↓	CTGF, NCX, SERCA2a, Collagen 1α1	Fibrosis, contractility
Farsangi. et al. (68)	*In vivo*	5 treadmill sessions/week for 4 weeks	Tissue	LncRNAs GAS5, MIAT, H19	H19↑, MIAT↓, GAS5↑	caspase-3	Fibrosis, apoptosis
Zhoua et al. (94)	Human, *in vitro*, *in vivo*	swimming training twice per day for 4 weeks	Venous blood, tissue	miR-222	miR-222↑	HIPK2, Bax/Bcl2, Caspase3/Caspase3 ratios, Collagen1	Apoptosis
Chen et al. (25)	Human, *in vitro*, *in vivo*	Swimming training twice a day for 4 weeks.	Plasma, tissue	Exosomal lncRNA CRNDE	CRNDE↑	miR-489-3p/Nrf2, Bax/Bcl-2, cleaved-caspase-3	Apoptosis, oxidative stress
Heiat et al. (52)	*In vivo*	5 treadmill sessions/week for 4 weeks	Heart blood, tissue	miR-126	miR-126↑	PGC-1α, TFAM, VEGF	Mitochondrial biogenesis, angiogenesis
Saati-Zarei et al. (57)	*In vivo*	3 treadmill sessions/week for 8 weeks	serum	miR-15a,miR-146a	miR-15a↓,miR-146a↓	VEGF, PI3 K, NOS	Angiogenesis
Liu et al. (93)	*In vivo*	5 treadmill sessions/week for 5 weeks	Tissue	miR-133a-3p	miR-133a-3p↓	CTGF, Bax, Bax/Bcl-2, Caspase 3, Cleaved Caspase-3	Apoptosis

### 4.1 miR-1 and miR-214

Electrical conduction, ion channel expression, and cardiac hypertrophy are regulated by miR-1, which is abundant in cardiac tissue. miR-1 is expressed at lower levels in cardiac hypertrophy. miR-1 has been linked to modulating the ACE2/Apelin pathway's actions. Apelin, a peptide hormone, binds to its receptor and activates signaling pathways that can affect cardiac function. Research has demonstrated that Apelin treatment increases miR-1 expression and reverses cardiac hypertrophy and impaired ventricular contractility caused by a high-fat diet (33). Moreover, miR-1 is believed to be anti-hypertrophic because it inhibits cardiomyocyte calcineurin/NFAT pathways via NFATc3 (34). The level of this protein is decreased in hypertrophic individuals and in rat cardiomyocytes treated with pro-hypertrophic stimuli, and its blockage is sufficient to induce cardiac hypertrophy (34). Heart failure and cardiac hypertrophy are cardiovascular disorders linked to miRNA-214 (35). Based on previous research, miRNA-214 can control the expression of genes related to heart remodeling and function. It was discovered in the aftermath of MI and can promote hypertrophy and cardiomyocyte fibrosis, which can lead to pathological cardiac remodeling (36).

In a study by Melo et al. (35), endurance training decreased myocardial hypertrophy and enhanced myocyte contractile performance in rats with myocardial infarction (35). Moreover, sarcoplasmic reticulum Ca2+-ATPase 2a (Serca-2a) and sarcolemma sodium/calcium exchanger (NCX) protein levels were elevated by the ET regimen. During the relaxation phase of the heart cycle, NCX removes calcium from cardiomyocytes, whereas the Na+/K+ ATPase pump actively transports sodium out of the cell. This helps maintain the appropriate ionic gradients and cellular conditions necessary for the subsequent contraction phase of the cardiac cycle. This exchange helps maintain the proper balance of calcium ions and ensures proper cardiac contractility. SERCA-2a is an essential protein involved in Ca2+ handling within cardiomyocytes. It is primarily located in the sarcoplasmic reticulum, a specialized organelle of muscle cells responsible for storing and releasing Ca2+ ions during muscle contraction and relaxation. Furthermore, exercise training enhanced the processes involved in the regulation and responsiveness of Ca2+ within rat cardiac muscle cells. Additionally, the study showed that exercise after MI partly restored miR-214 and miR-1 expression. Specifically, the exercise regimen targeted the restoration of Serca-2a levels to aid in the repair of miR-214, while focusing on NCX for partial miR-1 repair.

## 5 Cardiac angiogenesis in post-MI

The heart has an effective and dense microvascular network, making it a highly vascularized organ. Microcirculation is essential for providing the heart with oxygen and other nutrients, and functional and structural anomalies in microcirculation are linked to heart failure (HF). A sequence of events known as an ischemic attack results in the formation of new blood vessels that enhance the delivery of oxygen and nutrients. Therefore, the basic survival strategies for CAD include angiogenesis and arteriogenesis (37). A tightly controlled cascade of growth factors and paracrine mediators supports this process, which is directed by the coordinated actions of specialized endothelial cells (EC). In response to the VEGF gradient, a unique subset of ECs known as “tip cells” is formed. High mobility and specific protrusions at the leading edge of the sprouting vessel are two distinctive features of these tip cells. By expanding their filopodia and moving toward the VEGF gradient, tip cells are essential for initiating sprouting angiogenesis. However, in contrast to the tip cell region, “stalk cells” are found in the area of the VEGF gradient where the concentration is lower. The stalk cells generate the extended stalk of the developing vessel by trailing behind the tip cells (38, 39). The process involves the expression of a protein called apelin by newly created capillaries, which increases in response to ischemia. The expression levels of several angiogenic factors are known to change in response to overexpression of apelin in the recently established sprouting capillaries. Apelin-positive sprouting capillaries have higher mRNA levels of angiogenic factors than pre-existing collaterals. These genes include Cxcr-4 (a receptor for the chemokine stromal cell-derived factor-1), jagged canonical Notch ligand 1 (JAG1), hypoxia-inducible factor-1 (HIF-1), and VEGF receptor type 2. Cxcr-4 participates in endothelial progenitor cell recruitment and migration to ischemia sites. The JAG1 ligand coordinates the activation of the Notch signaling pathway, which promotes angiogenesis. The receptor for VEGF, a growth factor that facilitates angiogenesis and endothelial permeability, is called VEGF receptor type 2. Under low oxygen levels, the transcription factor HIF-1 becomes active and controls the expression of genes associated with angiogenesis. During repetitive ischemic insults involving inadequate blood supply to tissues or organs, the Apelin-positive sprouting collaterals adapt and respond to ongoing ischemic stress. Together with growing in size, these collaterals also develop due to the inclusion of smooth muscle cells (40). As a result, these collaterals undergo remodeling and expansion, increasing their size and blood-transport capacity. Aging-related and disease-causing factors can stimulate angiogenesis among mature individuals. The continuation of vascular homeostasis is crucial for the appropriate restructuring and restoration of tissues affected by insufficient blood flow, and angiogenesis is a pivotal mechanism in regulating this balance.

Hypoxia is a significant stimulus for the onset of angiogenesis (41). Hypoxia-inducible factors (HIF) are the prominent participants in hypoxia-driven angiogenesis. Following early MI, specific miRNAs are expressed in inflammatory cells, endothelial cells, and cardiomyocytes. They may remain stable for a few weeks following MI (37). Proangiogenic genes such as VEGF are transcriptionally regulated by the hypoxia-inducible factors HIF-1α and HIF-2α, which are expressed in endothelial cells (38). It is noteworthy that HIF-induced genes are also important for inflammation processes. This suggests that angiogenesis and inflammation are interconnected processes involved in the remodeling of ischemic tissue. In the ischemic heart, angiogenesis is promoted by several growth factors. Thus, in the region of developing vessels and the viable border zone of myocardiocytes, respectively, there is a significant increase in VEGF and its receptor VEGFR2 (39). It has been discovered that VEGF-B may have protective properties. Pro-survival signals that guarantee the emergence and maturation of new blood vessels in the afflicted area are thought to be induced by it. Research has demonstrated that VEGF-B facilitates the growth and maintenance of new blood vessels by inhibiting endothelial and mural cells from undergoing apoptosis (40). It is believed that increased PDGF-A and PDGF-D synthesis close to the infarcted area aids in the recruitment and stimulation of many cell types, including smooth muscle cells and endothelial cells, which are necessary for the development of new blood vessels (36). Physiological cardiac hypertrophy was investigated in Akt-1-activated experimental models with improved angiogenesis, primarily generated by VEGF and angiopoietin-2; in contrast, pathological myocardial remodeling was linked to reduced coronary angiogenesis. These findings demonstrated that pathological VR and HF may be caused by an imbalance between angiogenesis and heart repair and that stimulating cardiac angiogenesis may be the best treatment course. Vascular endothelial cells (ECs) are the most common non-cardiomyocytes in the heart and are receiving more attention due to their role in maintaining homeostasis and guiding organ repair.

## 6 Exercise-induced miRNAs involved in angiogenesis in post-MI

It is commonly known that some miRNAs are controlled in hypoxic environments. Increased angiogenesis in the heart has been linked to the overexpression of certain miRNAs, including miR-126 and miR-222, when exercise is performed (Figure 1). This can lessen the chance of ischemia and the ensuing myocardial infarction by increasing blood flow and oxygen delivery to heart tissues (Table 1).

### 6.1 miR-126

Important phases of angiogenesis, such as endothelial cell proliferation, migration, and tube formation, are significantly aided by miR-126 (42). miR-126 directly suppresses two negative regulatory proteins, Sprouty-related protein 1 (SPRED1) and phosphoinositide-3 kinase regulatory subunit 2 (PIK3R2), to exert its functional effects. The regulatory subunit 2 of phosphoinositide 3-kinase (PI3 K), PIK3R2, is a component of the PI3 K/Akt/induced endothelial nitric oxide synthase (eNOS) signaling pathway, which is essential for controlling angiogenesis. miR-126 directly sets the target and suppresses PIK3R2, thereby suppressing its expression. Repressing PIK3R2 allows miR-126 to counteract the downregulation of the PI3 K pathway, which in turn increases the activation of the following signaling pathways that promote the migration, proliferation, and vessel formation of endothelial cells. PIK3R2 is a component of the crucial PI3 K/Akt/eNOS signaling pathway that controls angiogenesis (43). Phospholipid inositol 4, 5-bisphosphate (PIP2) is phosphorylated by PI3 K to generate phosphatidylinositol 3, 4, 5-trisphosphate (PIP3). PIK3R2 acts as a modulatory element of PI3 K in this process. PIP3 is an intracellular messenger that carries protein kinase B (Akt) to the plasma membrane. Consequently, mammalian targets of rapamycin complex 2 (mTORC2) and phosphoinositide-dependent kinase 1 (PDK1) phosphorylate and activate Akt. Particular phosphorylation pathways are employed by induced Akt to phosphorylate and induce eNOS. Induced eNOS generates nitric oxide (NO), a potent vasodilation agent and pro-angiogenic molecule. NO mediates angiogenesis by encouraging relaxation of vascular smooth muscle cells, augmenting vasodilation, and facilitating neovascularization. Another target of miR-126 is SPRED1. The receptor tyrosine kinase signaling pathway, which participates in angiogenesis, is downregulated by SPRED1. It has been demonstrated that SPRED1 mediates angiogenesis via the Ras/MAPK pathway (43). The Ras/MAPK pathway is adversely modulated by SPRED1. It functions by blocking Ras from inducing and signaling, in addition to downstream effector molecules such as ERK and Raf. miR-126 directly binds to SPRED1 mRNA and reduces its expression levels. By downregulating SPRED1, miR-126 removes its inhibitory effects on receptor tyrosine kinase signaling, thereby increasing the activation of pro-angiogenic pathways. By directly repressing PIK3R2 and SPRED1, miR-126 serves as a fine-tuner of angiogenesis (44). During myocardial ischemia, activating the protein kinase B/glycogen synthase kinase 3 β (AKT/GSK3β) signaling pathway is crucial for protecting the heart from damage. Exercise and ischemia are two examples of the many stimuli that may activate this system (45). Bone marrow-derived endothelial progenitor cells (EPCs) from mice with MI exhibit activation of the AKT/GSK3β pathway following four weeks of exercise, as reported by Guo et al. (46). Additionally, it is known that the alteration in miR-126 expression, which is connected to the activation of the AKT/GSK3β pathway in EPCs, facilitates angiogenesis after MI (46). The AKT/GSK3β pathway has been announced to regulate miR-126 expression, and this modulation contributes toward the augmentation of angiogenesis due to MI (46). An investigation of zebrafish has shown that abnormal blood vessel development and integrity occur when miR-126 is either lacking or its function is compromised (47). Research on the selective deletion of miR-126 in mice has revealed that its absence causes blood vessel abnormalities. Downregulation of miR-126 leads to impaired proliferation of endothelial cells (ECs). Downregulation prevents proper vascular expansion and angiogenesis during embryonic development.

Although miR-126 is consistently down-regulated in a variety of conditions, such as cardiovascular disorders, there is also evidence that it may be up-regulated in response to specific stimuli, such as exercise. In healthy individuals, circulating miR-126 expression noticeably increased after 4 h of cycling and a maximum symptom-limited exercise test, as reported by Uhlemann et al. After the maximum exertion test and after four hours of cycling, the study demonstrated that miR-126 expression was augmented by 2.1 and 4.6 times, respectively (48). According to research by Chen et al. (49), transplanting mesenchymal stem cells (MSCs) treated with miR-126 increased angiogenesis and heart function in mice with MI. The results showed that adding miR-126 to MSCs improved their ability to cure MI-induced heart damage. The findings suggest that the augmented cardiac function observed in the experimental model was caused partially by miR-126-mediated enhancement of angiogenesis.

In their study (50), Song et al. used a rat model of myocardial infarction (MI) to examine the function and mechanism of miR-126 and HIF-1α in exercise-induced myocardial angiogenesis. The current study provided new insights into MI treatment. The researchers used human umbilical vein endothelial cells (HUVECs) and a post-MI rat model in their experiments. The findings demonstrated that 4-week ET markedly decreased the expression of SPRED1 and PIK3R2 and elevated the expression of miR-126and HIF-1α. The effects of ET were somewhat mitigated by the suppression of HIF-1α. *In vitro* experiments revealed that HIF-1α induced the expression miR-126 in normoxic and hypoxic HUVEC environments. It was discovered that miR-126 functions via the PI3 K/AKT/eNOS and MAPK signaling pathways to contribute to tube formation in HUVECs under hypoxia. The researchers found that the expression of miR-126 is regulated by HIF-1α. Specifically, their results showed that HIF-1α acts upstream to induce the expression of miR-126. The PI3 K/AKT/eNOS and MAPK signaling cascade is activated, promoting angiogenesis and eventually improving cardiac function in patients with MI. Gui et al. (51) investigated the mechanism by which endothelial progenitor cells (EPCs) in MI mice are more angiogenic after exercise when administered soluble epoxide hydrolase inhibitors (sEHi).

The study results showed that exercise protected against MI and promoted angiogenesis. Epoxyeicosatrienoic acids (EETs), which are metabolized by cytochrome P450 enzymes, play a role in promoting angiogenesis. Nonetheless, EETs are susceptible to instability and hydrolysis by soluble epoxide hydrolase (sEH). By inhibiting sEH, EET levels can be increased, leading to enhanced angiogenesis. The researchers used a novel sEHi called TPPU to increase EET levels in mice with MI. Both exercise and TPPU therapy can enhance the expression of miR-126, an miRNA recognized for its regulatory role in angiogenesis. Additionally, the miR-126 target gene Spred1, which negatively affects angiogenesis, was downregulated by exercise. TPPU treatment improved the effects of exercise on Spred1 and miR-126 expression. The results also showed that the shielding effect of TPPU on EPC activity was neutralized by the overexpression of Spred1. Furthermore, inhibiting miR-126 through antagomiR-126 reversed the effects of TPPU on Spred1 expression. The ERK/p38 MAPK pathway has a limited influence on miR-126 overexpression in TPPU.

In their study, Guo et al. (46) investigated the cardioprotective effects of ET along with a soluble epoxide hydrolase inhibitor (TPPU) in mice that had experienced an MI. The current study investigated whether elevated EET levels following TPPU administration could enhance the beneficial effects of exercise and improve cardiac recovery. To conduct the study, C57BL/6 mice were fed TPPU for 1 week before MI was induced. After recovery, the mice were subjected to the recommended training regimen. This study assessed the expression of miR-126 and its target gene, Spred1, and the activities of endothelial progenitor cells (EPCs). The researchers also investigated the activity of the AKT/GSK3β signaling pathway. The results demonstrated that ET enhanced angiogenesis after MI and significantly increased serum EET levels. The researchers found that TPPU, in addition to improving cardiac function, also helped amplify the beneficial effects of endurance training by reducing the size of the infarcted area. Additionally, miR-126 expression and EPC function were increased by ET and TPPU, respectively. In addition, Spred1 expression was downregulated. TPPU administration activated the AKT/GSK3b signaling pathway to become active. The current study concluded that EETs may play a role in the cardioprotective effects of exercise training following AMI. TPPU increased EET levels and promoted angiogenesis in the ischemic region, thereby improving exercise-induced heart recovery. According to the study, a combined therapeutic approach using pharmaceutical agents like TPPU along with targeting exercise-induced mediators such as EETs could represent a potential treatment strategy for MI. In a study by Heiat et al. (52), the researchers investigated the effects of high-intensity interval training (HIIT) on heart tissue damage and mitochondrial function in male rats following MI. By encouraging mitochondrial biogenesis and triggering angiogenesis signaling pathways, this study aimed to determine whether HIIT exercise can shield the heart from MI. Four groups (MI, Sham, HIIT, and HIIT+MI) were formed by the researchers from 24 male rats. The HIIT and HIIT+MI groups underwent a 4-week HIIT exercise program consisting of 10 intervals of 1-min running with a 2-min rest between each interval. The intensity of the HIIT was adjusted weekly based on the maximum treadmill running speed of the rats. The findings demonstrated that PGC-1α, TFAM, and VEGF levels were considerably higher after four weeks of HIIT exercise in the MI, HIIT, and HIIT+MI groups than in the sham group. Furthermore, although not substantially, HIIT exercise increased miR-126 expression. According to these results, HIIT exercise may provide cardioprotective benefits by reducing cardiac tissue damage and increasing angiogenesis and mitochondrial biogenesis in the heart tissue. The study emphasizes the value of exercise, especially HIIT, in enhancing cardiovascular health and preventing myocardial infarction. This study sheds light on the molecular mechanisms underlying the protective effects of exercise on the heart and highlights the potential of HIIT as a preconditioning regimen to protect cardiac tissue.

### 6.2 miR-15a and miR-146a

MiR-15a has been found to limit angiogenesis by concentrating on significant pro-angiogenic factors in particular conditions and disease states (53–57). VEGF is one of the recognized targets of miR-15a, and this interaction is linked to its anti-angiogenic effects of miR-15a. Studies have revealed that miR-15a may attach directly to the 3′ untranslated region (UTR) of VEGF mRNA, causing the protein to be destroyed and suppressed. By reducing VEGF synthesis, miR-15a can stop angiogenesis (57, 58). In addition to VEGF, miR-15a can target other pro-angiogenic factors, such as FGF2, PDGFRA, and platelet-derived growth factor receptor alpha (PDGFRA). By altering the expression of these components, miR-15a can adversely regulate angiogenesis. For miR-15a to exert antiangiogenic effects, Tie2 is essential. Therefore, the inhibitory action of miR-15a on Tie2 is necessary for its antiangiogenic activity. Inhibition of Tie2 prevents the proangiogenic response to anti-miR-15a treatment (59). Research findings indicate that pre-miR-15a transfection of HUVECs decreases protein expression and increases Tie2 mRNA expression by binding to Tie2 mRNA and preventing RNA translation (59, 60). Ischemia induced an increase in miR-15a but did not affect the amount of Tie2 protein.

Research has demonstrated that in mice, Tie2 disruption hinders post-ischemic regeneration. The involvement of miR-146a in cardiovascular disease is a subject of debate and ongoing research, and its role is controversial. Evidence indicates a correlation between sunitinib-induced cardiac dysfunction and a substantial reduction in miR-146a expression. Similarly, a mutual relationship exists, with lower miR-146a levels noted in situations involving cardiac dysfunction (61). According to a study by Zhang et al. (62), a deficiency in endogenous miRNA-146a worsened myocardial ischemia-reperfusion injury (MIRI). Additionally, miRNA-146a levels were elevated early in MIRI, suggesting that miRNA-146a has a protective function in this process. In a model of acute MI, Fang et al.'s (63) investigation of rats revealed that injection endothelium stem cells dramatically decreased miR-146a levels, decreased myocardial cell death, and decreased the size of the infarcted region. Studies have shown that miR-146a is upregulated in MI tissue (57, 64). It has been discovered that miR-146a targets many angiogenesis-related genes. Research has demonstrated that miR-146a can directly target and control NRAS expression. In specific cellular environments, by binding to the mRNA of NRAS, miR-146a can prevent its expression and reduce the amount of NRAS protein produced (65). The NRAS gene encodes the protein NRAS, which belongs to the RAS family of small GTPases. NRAS is involved in transmitting signals from cell surface receptors to the cell nucleus, affecting several cellular activities, such as proliferation, angiogenesis, and survival. NRAS mRNA and protein levels were decreased in HUVECs due to miR-146a overexpression (60). In rat cardiac ECs, miR-146a was similarly shown to downregulate NRAS (65). In rat cardiac ECs, miR-146a was similarly shown to downregulate NRAS (65). Similarly, in an experimental model of choroidal neovascularization (CNV), the introduction of NRAS-targeting small interfering RNA (siRNA) through intravitreal injections led to a reduction in vascular density (65). Saati-Zarei et al. (57) investigated the roles of VEGF, PI3 K, eNOS, miRNA-15a, miRNA-146a, and VEGF in mediating angiogenesis in rats induced by MI.

The following MI induction, six groups of male Wistar rats were generated. For 8 weeks, the participants were divided into groups that underwent different interventions, such as aerobic resistance exercise and vitamin D supplementation, or a combination of both exercise and vitamin D supplementation. The results suggest that aerobic resistance training combined with vitamin D supplementation resulted in the greatest improvement in heart function after MI. Compared with the other groups, the group showed greater exercise capacity, maximum load test, fractional shortening, and ejection fraction. Additionally, angiogenesis was significantly increased in this group. In addition, there was an increase in cardiac VEGF, PI3 K, and eNOS expression in the group receiving concurrent therapy, and there was a substantial reduction in the levels of circulating miRNA-15a and miRNA-146a. According to the study results, the combination of vitamin D supplementation and aerobic resistance training decreased miRNA-15a and miRNA-146a levels. These changes, in turn, stimulate angiogenesis through the VEGF/PI3 K/eNOS pathway, ultimately improving heart function after MI. The relevance of the association between miR-26a expression and cardiovascular disease is currently being investigated.

## 7 Cardiac fibrosis in post-MI

The ventricular compliance of the heart is known to decrease due to MI because it causes angiogenesis, cell necrosis, and increased collagen deposition. ECM proteins overly deposited in fibrosis include fibronectins, collagen I, collagen III, fibrins, and elastin. Approximately 60%–70% of human heart cells are cardiac fibroblasts (CFs), which are the primary sources of ECM formation. Excessive CF activation during MI results in increased interstitial fibrosis. Surprisingly, it was believed that the control of several miRNAs influenced the development and course of cardiac fibrosis by controlling the overexpression of specific proteins associated with these diseases (66–69).

## 8 Exercise-induced miRNAs as anti-fibrosis in hearts post-MI

Given the molecular mechanisms by which miRNAs regulate fibrosis, altered signaling pathways are usually associated with the pathological process of fibrotic growth (Figure 1) (Table 1) (70).

### 8.1 miR-29a and miR-101a

MiR-101a and miR-29a are involved in controlling the expression of fibrosis-related proteins, such as collagen. MiR-29a, b, and c are the three parts of miR-29 that exert antifibrotic effects (69, 71). It is widely recognized that miR-29 inhibits collagen expression. miR-29 reduces collagen production by binding to the 3′ UTR of target mRNA molecules and inhibiting their translation into proteins. The tissue and illness settings can influence the expression of collagen genes, which are targeted by miR-29. In addition to Col1a1, Col5a3, and Col4a2, miR-29 targets the collagen genes Col3a1 and Col7a1 (71, 72). In healthy tissue, miR-29 expression is high; in fibrotic conditions in patients and animal models (71, 73), it is low. In addition to directly targeting collagen genes, miR-29b can suppress cardiac fibrosis by binding to the coding region of TGF-β1 exon 3. This change can affect the transcription and mRNA stability of TGF-β1, ultimately lowering TGF-β1 levels. It was established that miR-29 suppresses TGF-β-mediated fibrosis through its negative connection with TGF-β/Smad signaling (73). Through the Smad3-dependent pathway, TGF-β1 mediates fibrosis by downregulating miR-29 (73). Qin et al. (74) observed that TGFβ1 therapy downregulated miR-29a expression in fibroblasts and PTECs that were cultivated. This downregulation was mediated by the activation of Smad proteins, which are critical downstream effectors of TGFβ1 signaling. Studies have indicated that fibrosis and ECM deposition are significantly influenced by Smad3, a crucial mediator of TGF-β/Smad signaling. In the context of UUO-induced renal fibrosis, Smad3 knockout exerts protective effects by modulating miR-29a expression and ECM protein levels (74). Activated Smad proteins bound to the promoter region of miR-29a and inhibited its transcription, reducing the amount of miR-29a (74). miR-29a treatment led to the downregulation of TGF-β1/Smad signaling. By downregulating TGF-β1/Smad signaling, miR-29a attenuated the fibrotic response (54, 73). Overexpression of miR-29a causes TGF-β1 and its downstream mediators, including Smad proteins, to be less expressed. Downregulating TGF-β1/Smad signaling is associated with overexpression of Smad7, an inhibitory protein that opposes TGF-β1 signaling (74).

It has been reported that cardiac tissue from patients with heart disorders expresses less miR-101 (71, 75–78). Cardiac fibrosis is a result of increased collagen production and CF proliferation. CF cell proliferation and collagen synthesis are inhibited by overexpression of miR-101a/b (76, 79). Induction of autophagy in CF cells by miR-101a directly controls the synthesis of collagen. The breakdown of intracellular collagen is mediated by autophagy, which may be the fundamental mechanism. Collagen production is inhibited by miR-101a by downregulating collagen expression, and autophagy is increased to reduce the total amount of collagen released by fibroblasts. MiR-101a induces cardiac fibroblast cell death (79). Apoptosis occurs in CF due to miR-101a. The antifibrotic activity of miR-101 is partially mediated by c-Fos. The FOS gene encodes c-Fos, which forms transcription factor activator protein-1 upon dimerization with c-Jun (80, 81). It has been demonstrated that upregulating c-Fos by tumor necrosis factor-α increases TGF-β1 transcription, a crucial cytokine that fosters fibrosis (82). In CF tissues, there was a substantial suppression of TGF-β1 expression upon c-Fos knockdown via siRNA. Conversely, TGF-β1 expression and collagen protein synthesis were both increased by c-Fos forced overexpression. Furthermore, when c-Fos expression was directly changed, either by overexpression or silencing with siRNA, miR-101 lost its ability to affect fibrosis, indicating that c-Fos/TGF-β1 is a downstream element in the miR-101 signaling pathway. Early administration of an miR-101a mimic to rats prevented the activation of TGF-β signaling and counteracted the temporary loss of endogenous miR-101a, which in turn reduced chronic cardiac fibrosis and preserved left ventricular function (79). Intermittent aerobic exercise was assessed by Xiao et al. (77) for its effects on the expression of miR-29a and miR-101a, as well as on genes associated with fibrosis, including Smad2/3, fos, TGF-β, COL1A1, and COL3A1.

For MI mice, the researchers intermittently performed aerobic activity beginning in the second week following surgery and continuing throughout the ninth week. In the immediate proximity of the infarcted area within the left ventricle, the expression of genes associated with fibrosis and miRNAs was assessed. Cardiac function was evaluated using the heart coefficient (HC) and hemodynamic assay. The results showed that in rats with MI, intermittent aerobic exercise significantly improved cardiac function and prevented collagen deposition in the myocardial interstitium. According to the analyses, exercise increased the expression of miR-101a and miR-29a, while also blocking the TGF-β signaling pathway. In a different investigation, Meloa et al. (67) examined how swimming training affected the expression of collagen and miRNA-29 in MI rats. The study conducted swimming training sessions for 10 weeks starting 4 weeks after MI was induced in the rats. The infarcted area, border region, and distant myocardium of the left ventricle were the cardiac locations where the researchers examined the expression of collagen and miRNA-29. The findings demonstrated that compared with sedentary rats with MI, swimming training enhanced miRNA-29c and miRNA-29a expression in the border area and distant myocardium. Compared with the sedentary group, the swimming-trained group exhibited reduced expression of collagen types I and III. These results imply that swimming exercise can lower collagen expression and increase miRNA-29 levels in particular heart areas during MI, which may help improve ventricular function.

## 9 Exercise-induced lncRNAs as anti-fibrosis in hearts post-MI

LncRNAs are considered viable therapeutic targets for CVDs because of their aberrant expression in cardiac illnesses (Table 1).

### 9.1 LncRNA H19

After MI, increased H19 levels have been observed in the heart (83). According to Choong et al. (83), H19 modulates the ECM, which promotes fibrotic activity during the early stages of cardiac remodeling after MI. Heart fibroblasts at the infarct border zone exhibit higher H19 levels than cardiac cardiomyocytes in the early stages after MI. H19 attaches to Y-box binding protein 1 (YB-1), preventing YB-1 from inhibiting collagen production. Zhou et al. (84) investigated the function of H19 in terms of MI in two separate investigations. In their first study, Zhou et al. found that the expression of long noncoding RNA H19 decreased 3 weeks after MI. This discovery implies that H19 may have a dynamic expression pattern at various post-MI stages. H19 may have been involved in the early or acute phase of heart damage and remodeling, as suggested by the downregulation of H19 at that time. In a second study by Zhou et al., overexpressing H19 using lentivirus carrying the pcDNA-H19 construct increased the levels of H19 in cardiac tissue. The upregulation of beclin-1 and autophagy-related genes (Atgs) under these conditions suggested that H19 overexpression was linked to the induction of autophagy. Improved heart function and decreased infarcted area were the outcomes of H19 overexpression-induced autophagy upregulation (84). Farsangi et al. (68) noted that the reintroduction of exercise reduced H19 expression to baseline levels. Exercise appears to reduce fibrosis and enhance cardiac function by partially modulating H19 expression, which is reasonable considering that H19 plays a pathophysiological role in the mechanisms that lead to cardiac remodeling, especially in fibrosis.

### 9.2 LncRNA MIAT

Additionally, it was demonstrated that myocardial ischemia/reperfusion damage elevated MIAT expression (85). In the pathophysiology of MI, MIAT has been identified as a pro-fibrotic lncRNA (86). The peri-infarct region experiences an increase in MIAT expression, which triggers cardiac fibrosis via the MIAT/miR-24/Furin axis (86). miR-24 inhibits TGF-β signaling, which is activated by furin. After MI, MIAT counteracts the inhibitory effect of miR24 on Furin, which leads to the onset of cardiac fibrosis. Exercise treatment may reduce heart fibrosis by decreasing MIAT expression (68).

### 9.3 LncRNA GAS5

The well-known lncRNA GAS5 controls the development of fibrosis in MI and the death of cardiomyocytes (87). Moreover, conflicting results from several studies suggest that the function of GAS5 (Growth Arrest-Specific 5) in cardiomyocyte apoptosis in MI is complicated. In a study by Zhang et al. (88), they demonstrated that in an isoproterenol (ISO)-induced MI model, siRNA-mediated suppression of lncRNA GAS5 decreased cardiomyocyte death and improved subsequent cardiac fibrosis. This suggests that suppressing GAS5 expression might be protective and that elevated GAS5 levels during MI might encourage cardiomyocyte death. However, in a different investigation, Zhang et al. discovered that elevated GAS5 levels in cultured MI cells promoted cardiomyocyte death. This result supports the findings of a previous study showing that GAS5 exerts pro-apoptotic effects. However, Hao et al. (89) reported different outcomes. Induction of GAS5 expression inhibited cardiomyocyte apoptosis in a rat left anterior descending (LAD) model of MI. It was suggested that the anti-apoptotic properties of GAS5 are achieved by inhibiting the apoptotic factor sema3a in cardiomyocytes. GAS5 could affect cardiomyocyte apoptosis differently depending on the circumstances, which could be affected by the length and intensity of ischemia, the stage of MI, or the existence of additional interacting molecules. According to the findings of Farsangi et al. (68) GAS5 expression in MI mice was markedly elevated upon treatment with Ex. Nonetheless, the fact that their model's elevated GAS5 expression was linked to beneficial effects on post-MI pathogenesis is consistent with Hao et al.'s findings.

## 10 Cardiac apoptosis in post-MI

Apoptosis is a specific form of programed cell death characterized by several distinctive morphological features. These processes include cell contracting, fragmentation into membrane-bound apoptotic bodies, and prompt phagocytosis by neighboring cells without triggering an inflammatory response (53). Cardiomyocyte apoptosis, which occurs in the context of MI, leads to abnormal cardiac remodeling and reduced ventricular compliance (48, 54, 55). Previous studies have demonstrated that miRNAs influence apoptosis, a crucial aspect of MI. In MI, downregulation of miR-17-5p improves heart function by preventing endothelial cell death (56). Upregulating miR-298 augmented infarcted heart performance and dramatically diminished the expression of cleaved caspase-3, BAX, and cytochrome c in a rat model of infarction. Additionally, it diminished myocardial apoptosis (90).

## 11 Exercise-induced miRNAs as anti-apoptosis in hearts post-MI

In MI, exercise-induced miRNAs have been identified as putative regulators of several cellular processes, including apoptosis (Figure 1). It has been discovered that several exercise-induced miRNAs have anti-apoptotic characteristics in the heart following MI. For example, miR-497 (91), miR-1192 (92), miR-133a (93), and miR-222 (94) are upregulated in response to exercise and exert protective effects against apoptosis (Table 1). These miRNAs can target and regulate the expression of specific genes involved in apoptotic signaling pathways, such as BAX, caspases, and p53. By either inhibiting pro-apoptotic gene expression or promoting anti-apoptotic gene expression, exercise-induced miRNAs can modulate apoptotic signaling pathways in the heart. This allows cells to enhance their survival and reduce tissue damage.

### 11.1 miR-133a

miR-133a is highly expressed in healthy, well-functioning hearts. miR-133a undergoes significant changes in response to cardiac injury and is essential for healing following acute MI. Studies have shown that miR-133 protects the heart by reducing the number of myocardial infarcts, cardiac damage, oxidative stress, inflammation, and cardiomyocyte death by inhibiting the SIRT3/AMPK pathway (95). Liu et al. (93) reported that miR-133a-3p regulates connective tissue growth factor (CTGF) production, which is linked to cardiomyocyte apoptosis and improved cardiac function. CTGF is a member of the stromal cell protein family CCN. It is a 38 kDa protein that is composed of 5 exons. It is induced by the extracellular matrix and is highly evolutionarily conserved across vertebrates (96). CTGF is a cardiac autocrine factor that regulates several essential biological processes, including fibrosis, apoptosis, and angiogenesis, among other pathological processes, in various heart diseases (97–99). The researchers carried out experiments involving male mice divided into several groups, including sham-operated, sham-operated with aerobic exercise, MI, and MI with aerobic exercise. The findings revealed that in comparison with the sham group, the MI group exhibited reduced miR-133a-3p and Bcl-2 levels, compromised cardiac function, and elevated CTGF, Bax, Bax/Bcl-2 ratio, Caspase 3, and cleaved caspase 3. On the other hand, the MI group that exercised aerobically showed reduced levels of CTGF, Caspase 3, Cleaved Caspase-3, Bax, and Bax/Bcl-2, enhanced cardiac function, and elevated miR-133a-3p and Bcl-2 levels. Additional research that confirmed the connection between miR-133a-3p, CTGF, and cardiac function was conducted on rat cardiomyocytes using H2O2 and AICAR. Based on their study, the scientists concluded that aerobic exercise can enhance cardiac function by preventing cardiomyocyte apoptosis and downregulating CTGF expression in the myocardium of MI mice via miR-133a-3p.

### 11.2 miR-1192

C2C12, cardiac, and muscle cells express miR-1192. MiR-1192 regulates myogenesis by altering the translation of HMGB1 mRNA (100). miR-1192 inhibits the production of heparin-binding EGF-like growth factor (HB-EGF), and it also targets IL-17 to affect T cell development and osteogenic differentiation (101, 102). A 4-week swim training program improved heart function and decreased cardiac apoptosis in mice, as reported by Wang et al. (92), and showed protective benefits against MI. Additional research showed that miR-1192 significantly protected cultured newborn cardiomyocytes against hypoxia, and plasma miRNA profiling showed that miR-1192 was elevated during exercise. The researchers also performed intramyocardial agomiR-1192 injection, which mimicked the effects of exercise-induced upregulation, and observed similar cardioprotective effects. On the other hand, in MI mice, the cardioprotective effect of exercise was eliminated by blocking miR-1192 with antagomiR-1192. According to the study, miR-1192 is upregulated during exercise and shields the heart from MI. Furthermore, it was shown that miR-1192 targets caspase 3 in cardiomyocytes to provide cardioprotective function.

### 11.3 miR-497

A variety of cancer types are suppressed by miR-497 (103–106). Research has demonstrated that miR-497 is a potential MI diagnostic marker. A reliable association was found between human miR-497 and acute MI (107). According to Li et al. (108), blocking miR-497 diminished the impact of anoxia and reoxygenation damage in cardiomyocytes by facilitating autophagy and preventing cell death. The suppression of miR-497 through activation of the Wnt/β-catenin signaling pathway is a critical component of bone marrow mesenchymal stem cell (BMSC) transplantation during MI treatment (108). Li et al. conducted a study to explore whether miR-497 regulation is implicated in the beneficial effects of ET on MI. According to the study results, ET decreased the size of the infarcted region in MI mice and suppressed miR-497 expression. The incidence of an infarcted region increased after the injection of the miR-497 agomir, but the miR-497 antagomir amplifies the beneficial effects of ET on MI. According to the study, the most likely target of miR-497 is chloride voltage-gated channel 3 (CLCN3). Furthermore, improved MI through ET was related to the regulation of apoptotic and inflammatory factors. The processes underlying the beneficial effects of ET on MI are closely associated with miR-497 control. Overall, the research revealed that blocking miR-497 can increase the protective effects of ET against myocardial infarction. The processes underlying post-MI cardiac rehabilitation may become more evident with a better understanding of miR-497 control and its role in ventricular remodeling.

### 11.4 miR-222

Studies have shown that by lowering cell death and increasing cell survival, miR-222 can preserve cardiac muscle cells during ischemia. This process occurs through the targeting of specific genes involved in apoptosis and cellular stress responses. Studies have shown evidence that poorer prognosis in mice with experimental MI is associated with decreased blood miR-222 levels (94). In particular, previous studies have provided evidence that MI patients who experience readmission and/or death have significantly diminished serum miR-222 levels than those with more favorable outcomes (94). Direct interactions between miR-222 and HIPK2 mRNA have been reported, leading to the downregulation of HIPK2 protein levels (109, 110). Downregulation of HIPK2 expression by miR-222 may affect HIPK2-regulated cellular processes and signaling pathways regulated by HIPK2. Many pathways have been identified as being influenced by HIPK2, among them the p53 pathway, which is crucial for regulating cell cycle arrest and programed cell death in response to DNA damage. By targeting HIPK2, miR-222 can influence these pathways and affect cell outcomes (113–115). HIPK2 inhibition reduces P53 phosphorylation and attenuates cardiomyocyte death and MI (94). Zhoua et al. (94) reported that exercise decreased HIPK2 levels, whereas MI increased HIPK2 levels. *In vitro* studies employing cardiomyocytes and animal models have demonstrated that HIPK2 inhibition inhibits the apoptotic response to oxygen glucose deprivation/reperfusion (OGD/R). Furthermore, after acute MI, HIPK2 suppression decreased the extent of heart tissue damage and maintained cardiac function throughout post-MI remodeling. The results also revealed that the protective effects of HIPK2 suppression on apoptosis are mediated by P53 phosphorylation. In addition, elevated miR-222 levels, a transcription factor that targets HIPK2, protected against cardiac dysfunction after MI. Compared with healthy individuals, patients with MI had considerably lower serum miR-222 levels, suggesting that miR-222 could be a prognostic biomarker. These results imply that by lowering P-P53 levels, exercise-induced HIPK2 suppression might mitigate cardiomyocyte apoptosis and shield against MI. Inhibiting HIPK2 is a potential therapeutic intervention for MI and post-MI heart failure. The current study emphasized the potential of ET to improve heart remodeling and function after AMI.

## 12 Extracellular vesicles (EVs): from biogenesis to uptake

**Figure 2:**
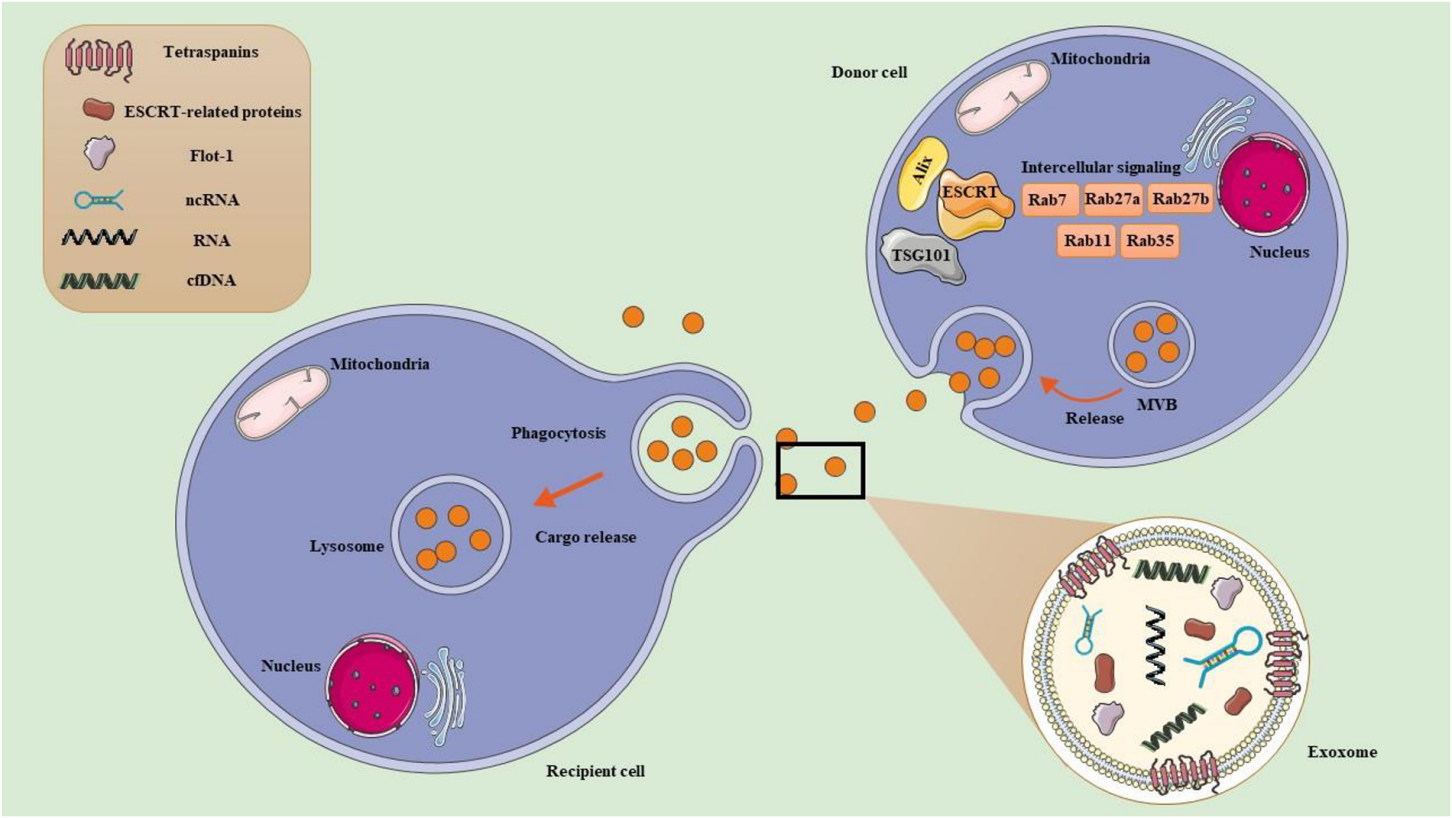
Exercise-derived extracellular vesicles (EVs). (1) Exosome formation. Early endosomes originate from the inward budding of the plasma membrane. They mature into late endosomes, which are characterized by the presence of multiple intraluminal vesicles (ILVs). Late endosomes can also be referred to as multivesicular bodies (MVBs) due to their ILV content. MVBs exist in different subpopulations and play critical roles in cellular transport. MVBs can either fuse with lysosomes for degradation, or fuse with the PM to release ILVs into the extracellular space as exosome. MVB development can take place through two main pathways: ESCRT-dependent pathways, which involve the Endosomal Sorting Complex Required for Transport, and ESCRT-independent pathways, which include tetraspanins and ceramides. Additionally, Rab GTPases (RABs) play a role in regulating the sorting and transport of exosomes, while SNARE proteins, known as soluble N-ethylmaleimide sensitive factor attachment protein receptors, facilitate the processes of membrane fusion. (2) Exosome uptake in recipient cell. Once released, exosomes can enter recipient cells either by directly binding to the plasma membrane or through engulfment. Their uptake can happen via various mechanisms, including endocytosis (such as phagocytosis, micropinocytosis, clathrin-mediated endocytosis, caveolin-mediated endocytosis, and receptor-mediated endocytosis) or by directly fusing with the recipient cell's plasma membrane. After binding, the cargo from the exosome of the donor cell is delivered into the recipient cell.

EVs serve as additional means of intercellular communication (Figure 2). EVs are vesicular structures enclosing lipid membranes. It is believed that these structures transport a variety of biological cargo, including proteins, nucleic acids (such as RNA), and lipids (114–117). It is postulated that these payloads are delivered to distant and local recipient cells and may impact recipient cell activity in an endocrine or juxtacrine manner, respectively (118). The three primary classifications of EVs are exosomes, MVs, and apoptotic bodies (118). These EV subtypes are characterized by their size and the processes involved in their formation. Apoptotic bodies are the largest type of extracellular vesicles, ranging from approximately 500 to 4,000 nm in size, and they are produced as a result of programmed cell death. A cell undergoing apoptosis goes through several stages, ultimately leading to the breakdown of cellular components contained within separate membrane-bound vesicles known as apoptotic bodies. As a result, they are typically characterized by the presence of organelles and/or nuclear material within their lumen. Although they are not usually linked to extracellular vesicles, unconventional secretion processes like lysosomal vesicle secretion and secretory autophagy can release various cytoplasmic substrates into the extracellular space under certain conditions, potentially through mechanisms that are similar to, yet distinct from, those of other EVs (119). MVs, also referred to as ectosomes, microparticles, or shedding vesicles, are small membrane-bound vesicles ranging in size from approximately 100 to 1,000 nm. They are formed through the outward budding of the plasma membrane, a process that allows cells to release these vesicles into the extracellular space. MVs play various roles in intercellular communication and can transport proteins, lipids, and nucleic acids, influencing numerous physiological and pathological processes. MVs are differentiated from apoptotic bodies not only by their size, which typically ranges from 100 to 1,000 nm compared to the larger size of apoptotic bodies that can reach up to 5,000 nm, but also by their distinct formation process, as MVs bud directly from the plasma membrane while apoptotic bodies result from the collapse and fragmentation of cells undergoing programmed cell death (118). Furthermore, MVs are characterized by unique membrane-specific antigens that help identify their parent cells, enhancing our understanding of their function in physiological and pathological contexts, in stark contrast to the more heterogeneous composition of apoptotic bodies (120). Exosomes are the smallest type of extracellular vesicles, measuring approximately 40–120 nm in diameter. They are formed through a complex process that involves the inward budding of endosomes, leading to the creation of multivesicular bodies (MVBs). These MVBs can subsequently fuse with the plasma membrane, releasing exosomes into the extracellular space. This biogenetic pathway distinguishes exosomes from larger vesicles like MVs, highlighting their unique role in cellular communication and signaling (121).

As part of normal and abnormal processes, almost all cells can release exosome. Exosome biogenesis often occurs with the endocytosis of extracellular materials, which generate early endosomes. Early endosomes ultimately develop into late endosomes through interactions with various intracellular vesicles and organelles, such as the Golgi complex and endoplasmic reticulum. The continuous inward folding of the membrane in late endosomes contributes to the generation of MVBs, which efficiently transport exosomes. MVBs are directed toward and integrate with the plasma membrane through collaboration with the Rab family of GTPase proteins, which ultimately discharge exosomes into the extracellular milieu (122). The exosomes are released from the donor cell when they are discharged and either fuse with the plasma membrane or are absorbed by the destination cells (21). Thus, they have an impact on regulating recipient cells' physiological and pathological responses (123). Exosomes in biological fluids can offer a window into altered cellular or tissue states in various diseases, potentially serving as multimodal diagnostic readouts.

Recent studies have indicated that after exercise, there is a notable increase in the levels of EVs along with their associated miRNAs and lncRNAs (Figure 2). This rise is significant as it highlights the role of EVs in cellular communication and adaptation in response to physical activity (118). Exercise stimulates muscle cells and other tissues to release EVs (124–126), which can be triggered by mechanical stress, metabolic changes, or activated signaling pathways during physical activity (127). This activity can also modify the expression of specific miRNAs and lncRNAs, contributing to the body's adaptive responses that enhance muscle repair, growth, and metabolic regulation. EVs act as carriers, delivering ncRNAs to target cells, enabling these ncRNAs to regulate gene expression in distant tissues. The ncRNAs within ELVs can affect various signaling pathways related to inflammation, cell signaling, and muscle regeneration (25). Furthermore, the lncRNAs and miRNAs released in EVs can modulate gene expression in recipient cells, fostering beneficial adaptations like improved muscle recovery and anti-inflammatory responses post-exercise (25). Exercise can induce mild stress and hypoxia (127), which may further boost the production and release of EVs and their contents (127). Additionally, hormones released during exercise, such as catecholamines and growth factors (128), can also promote the release of EVs and their cargo.

## 13 Exercise-derived exosomes in post-MI

Exercise-derived exosomes have emerged as a significant focus of research in the context of post-MI recovery (129). These exosomes play crucial roles in cardiac healing and regeneration after an MI event (Table 2). This approach could provide new strategies for managing cardiomyopathy. Chen et al. (25) investigated the role of exosomal lncRNAs in MI and the potential therapeutic effects of exosomes derived from exercise. The specific lncRNA investigated in this study was CRNDE, which was found in exosomes generated during prolonged exercise training. They found that exosomes produced from exercise had high CRNDE expression. They found that decreasing CRNDE levels slowed the MI course and enhanced cardiomyocyte apoptosis and oxidative stress. It was discovered that the transcription factor Nrf2, which is essential for cellular defense against oxidative stress (130), is expressed differently when CRNDE functions as a sponge for miR-489-3p (25). In addition, in hypoxic cardiomyocytes, inhibiting miR-489-3p successfully reversed the effects of CRNDE depletion. These results provide a prospective therapeutic alternative for MI therapy by indicating that the long-term exercise-derived exosomal lncRNA CRNDE contributes to mitigating MI damage through the miR-489-3p/Nrf2 signaling axis. This research can help develop treatment approaches and help construct a national fitness public service system by clarifying the processes behind their impacts on MI.

**Table 2:** Role of exercise-derived exosomal non-coding RNAs in hearts post-MI.

Cargo	Type of models	Methodology of exercise	Sample	Expression after exercise	Target genes	Modulation	Ref
LncRNA CRNDE	Human, *in vivo*, *in vitro*	Swimming twice a day for 4 weeks	Plasma, tissue	CRNDE↑	miR-489-3p/Nrf2	Apoptosis ↓, oxidative stress↓	(25)

## 14 Conclusions

Emerging evidence indicates that exercise protects against MI by altering noncoding RNAs, specifically miRNAs. Exercise-induced miRNAs in post-MI heart tissue are essential for controlling several cellular functions, including apoptosis. It was discovered that miRNAs generated during exercise may regulate the heart's apoptotic processes. These miRNAs diminish tissue damage and increase cell survival by targeting pro-apoptotic genes or encouraging the production of anti-apoptotic genes. In addition to their direct cardioprotective effects, miRNAs produced during exercise can regulate a variety of other important cellular processes, including angiogenesis, inflammation, and the oxidative stress response. These activities are directly linked to post-MI apoptosis and cardiac remodeling. Exercise-induced miRNAs contribute to the cardioprotective effects of exercise via several methods. The discovery that exercise-induced miRNAs are essential regulators of MI shed light on the molecular processes underlying the heart-healthy effects of exercise. These findings have significant implications for developing novel therapeutic approaches that harness the cardioprotective effects of exercise-induced miRNAs.
